# A dogs-at-work program in a veterinary college: promoting workplace wellbeing and resilience

**DOI:** 10.3389/fpsyg.2026.1768459

**Published:** 2026-03-26

**Authors:** Emilie M. MacInnis, Niwako Ogata, Leanne O. Nieforth

**Affiliations:** 1 Department of Comparative Pathobiology, Human-Animal Partnerships and Interactions Lab, Center for the Human-Animal Bond, College of Veterinary Medicine, Purdue University, West Lafayette, IN, United States; 2 Department of Veterinary Clinical Sciences, Center for the Human-Animal Bond, College of Veterinary Medicine, Purdue University, West Lafayette, IN, United States

**Keywords:** companion animal, human-animal, veterinary staff, workplace resilience, workplace wellbeing

## Abstract

Dogs-at-work (DAW) programs may be able to take advantage of the benefits of human-animal interactions to support the resilience and wellbeing of employees in high stress workplaces, but research in this area is limited. Consequently, employers are often hesitant to implement these programs. To expand our understanding and support implementation of these programs, this study uses the Resilience Portfolio Model to explore how wellbeing and resilience differ between veterinary staff, based on participation in a long-standing dogs-at-work program. An online survey consisting of open-ended questions about participants’ perceptions and experiences with the program, and validated measures of mental health and work-related wellbeing, was distributed to faculty and staff eligible to participate in the DAW program. Results demonstrated that the 23 DAW participants and 65 non-participants were comparable across nearly all wellbeing measures, in alignment with population norms. Qualitative coding revealed four themes for perceptions and experiences with the DAW program and participating dogs: (1) an increase in social interactions, (2) improvements in mental health, (3) opportunities for physical activity, and (4) recommendations for program logistics. In line with the Resilience Portfolio Model, meaning-making, regulatory, interpersonal, and environmental strengths were all represented in the findings. Taken together, results indicated that veterinary staff perceived benefits from interacting with dogs at work and may draw resilience-supporting strengths from these interactions. Results also highlighted practical opportunities for program improvement, including equitable access and long-term program management needs.

## Introduction

Employees in the veterinary field have been found to experience high levels of burnout and intention to leave the field (Kogan and Rishniw, 2024; Takefuji, 2025). Additionally, the veterinary field is experiencing a staffing shortage (AAVMC, 2022), and estimates indicate that this shortage of veterinarians and other veterinary staff will worsen by 2030 (Lloyd, 2021, 2023). Therefore, understanding programs that may support wellbeing and resilience in the veterinary field is crucial to foster and maintain a healthy, adequately staffed veterinary workforce. Programs that allow staff to bring their dogs to work, henceforth referred to as Dogs-At-Work (DAW) programs, may be one low-cost option to support the wellbeing of veterinary staff. While veterinary staff often interact with multiple dogs a day, these dogs are typically patients experiencing mild to severe distress. Interacting regularly with these patients is one contributor to the high levels of stress in the veterinary field (O’Connor, 2019). Therefore, it is important to understand the experiences of veterinary staff in the context of dogs as companions in the workplace.

Research shows that potential benefits from interacting with dogs include companionship, emotional and social support, decreased loneliness, lowered anxiety levels, and better mental and physical health (Fine et al., 2025). It may be possible for employers to bring these benefits into the workplace via DAW programs. DAW programs allow employees to bring their companion dogs to their workplace on a regular basis (Kelemen et al., 2020). Previous studies have demonstrated that both managers and employees perceive benefits from the presence of dogs in the workplace (Sousa et al., 2022; Junça-Silva and Galrito, 2024). The presence of dogs in the workplace appears to support workplace engagement and employer commitment, and lower employee stress levels (Barker et al., 2012; Junça-Silva, 2022; Sousa et al., 2022). Despite this, managers in companies without DAW programs may be hesitant to implement these programs, citing a lack of research and concerns with practical challenges (Junça-Silva and Galrito, 2024). Potential challenges faced when integrating dogs into the workplace include: accommodating employees that dislike or fear dogs, accommodating employees that are allergic, facing perceptions of unfair treatment for those who cannot participate, avoiding or dealing with dog bites, addressing animal welfare needs, and accounting for budgetary demands (Foreman et al., 2017; Junça-Silva and Galrito, 2024). Considering that the ability to successfully and safely implement a DAW program is highly dependent on individual work environments and employers, it is important to develop a thorough understanding of the experiences of, and effects on, employees in workplaces with DAW programs. This understanding can support employers and managers interested in implementing these programs in confidently making informed decisions. Thus, this exploratory study examined a DAW program within a veterinary college. This DAW program is a multi-decade program established and continuously run by veterinary behaviorists. This program presents a unique opportunity to explore a well-established DAW program in high-stress, animal-friendly workplace.

### Theoretical framework

The Resilience Portfolio Model (RPM) is a strengths-based framework that characterizes wellbeing outcomes following exposure to trauma or other adverse experiences as influenced by protective factors (Grych et al., 2015). Veterinary staff are at high risk for consistent exposure to traumatic events, both directly (administering euthanasia, risk of bites, etc.) and indirectly (treating injuries, supporting bereaved clients, handling financial concerns, etc.) (Hanrahan et al., 2018). While these traumatic events are an inherent aspect of working in veterinary medicine, exposure to these events is correlated with higher levels of burnout and stress (Ouedraogo et al., 2021). The RPM may provide a framework to understand and enhance wellbeing outcomes via protective factors. Protective factors are the modifiable strengths that a person does or does not have access to, as well as the response a person has to the adverse experience (Grych et al., 2015). These strengths, which include internal assets and external resources, make up the unique Resilience Portfolio of an individual (Banyard et al., 2025). Within the RPM, all strengths fall into one of four domains: (1) meaning-making strengths, or how an individual makes sense of life events and maintains coherence between those events and their personal beliefs and values; (2) regulatory strengths, which allow an individual to support or sustain goal-driven behavior; (3) interpersonal strengths, or how an individual creates and maintains close friendships, romantic relationships, and familial connections; and (4) environmental strengths, which are aspects of both natural and human-made environments that promote resilience, such as access to green spaces, supportive workplace policies, or feeling safe in one’s neighborhood (Grych et al., 2015; Banyard et al., 2025). RPM can be used to understand what tools an individual has to cope with adverse events and how they may or may not struggle amidst ongoing stressors, such as employment in a high-demand workplace.

#### Resilience portfolio model in the workplace

The RPM has been used to examine workplace wellbeing for employees in high-stress roles including doctors, emergency workers, and social workers (Wachter et al., 2020; Whittenbury et al., 2025). While the veterinary field has yet to be examined through the lens of the RPM, it is similar to these high-stress, caregiving career fields. Therefore, the RPM may be able to give insight into strengths that veterinary staff draw from to support resilience and wellbeing. Studies on RPM in the workplace found that employees draw upon many aspects of all four domains of strength (meaning-making, regulatory, interpersonal, and environmental) to navigate stressful workplaces.

Previous research on resilience in the workplace conceptualized workplace strengths as the fit between an individual and their workplace (Wachter et al., 2020). Drawing on that conceptualization, the current study utilized both a measure of work-related quality of life and a measure of work engagement to characterize general workplace resilience and the meaning-making strength within the RPM framework. Work-related quality of life is conceptualized as a full picture of work and non-work factors that influence how an individual approaches, and is impacted by, work (Van Laar et al., 2007). Work engagement is defined as “a sense of energetic and effective connection with…work activities” (Schaufeli and Bakker, 2004). Measuring an individual’s approach to work, the impact of that work, and their engagement with work can characterize the fit between an individual and their workplace broadly, in line with Wachter et al.’s (2020) conceptualization of workplace resilience.

#### Dogs and the resilience portfolio model

When employers intentionally designed supportive and safe workspaces, employees in these high-stress roles demonstrated greater resilience and more effective responses to workplace stress (Wachter et al., 2020; Whittenbury et al., 2025). One intentional choice employers may implement to foster resilience and wellbeing is a DAW program. Dogs have the possibility to fall within multiple RPM domains. They have been shown to support the first strength, meaning-making, by fostering a sense of identity, self-worth, and purpose (Brooks et al., 2018). Additionally, interacting with dogs lowers physiological and psychological indicators of stress (Applebaum et al., 2021). Thus, the second strength, the regulatory strength, may be supported by choosing to interact with a dog in a high-stress scenario (Whittenbury et al., 2025). Additionally, physical activity is a known regulatory strength, and dogs have been shown to increase physical activity for their household (McConnell et al., 2011). The presence of the third strength, interpersonal strength, is often indicated by strong, supportive familial relationships and dogs are often considered significant members of the family (Volsche, 2019; Banyard et al., 2025). Thus, strong bonds with a dog may promote resilience and may be a marker of interpersonal strength (Applebaum et al., 2021; Banyard et al., 2025). Dogs may also serve as an environmental strength, the fourth strength in the RPM, by making an individual feel safe and comforted in their environment (Banyard et al., 2025; Whittenbury et al., 2025). Therefore, DAW programs have the potential to support resilience and employee wellbeing by bringing the meaning-making, regulatory, interpersonal, and environmental strengths of dogs into the workplace.

The veterinary field is well-positioned to incorporate DAW programs, as the veterinary workplace is typically already dog-friendly and dog-safe, and many of the common challenges to implementing such programs, such as fear of animals and allergies, are likely not present for these employees. Considering the high levels of burnout in this field, veterinary staff stand to gain much from novel programs aimed at promoting resilience and wellbeing.

### Current study

Despite the need to support the wellbeing of veterinary staff and the potential benefits of DAW programs, to date no study has examined DAW programs or workplace wellbeing in the veterinary field via the RPM. This cross-sectional, exploratory study utilized a convergent parallel mixed-methods design to expand on the workplace wellbeing and resilience literature. The overarching objective of this study was to explore how workplace wellbeing and resilience differs between staff members of a veterinary college, based on participating or not participating in the long-standing DAW program offered at this college. The RPM framework was used to conceptualize the personal and workplace strengths supporting wellbeing and resilience, as well as the measurable outcomes tied to wellbeing and resilience. Thus, the research question for this study was “How does resilience and workplace wellbeing differ between staff of a midwestern veterinary college, based on participation in a long-standing Dogs-At-Work program?” In addition to employee wellbeing and resilience, it is crucial to thoroughly understand the practical logistics and potential challenges of DAW programs. This will allow employers to ensure that employees and their dogs are safe, healthy, and supported. Thus, both theoretical and practical implications of the DAW program are discussed.

## Methods

### Procedures

This study was approved as an exempt study by the University Institutional Review Board (IRB #2024-196). A cross-sectional mixed methods survey was employed to evaluate the outcomes of a DAW program at a veterinary school within a large Midwestern university. The survey included questions about the respondent’s role and work experiences, validated measures of wellbeing, and open-ended short response questions about the respondent’s knowledge of and experience with the DAW program.

#### Dogs-at-work program background

This program is an optional employment benefit that allows veterinary school staff and faculty to bring their own pet dogs to work with them. A veterinarian and faculty member at the veterinary school implemented the DAW program in the mid-1990s, and the program was continued by their successor, who will henceforth be referred to as the DAW supervisor. The current DAW supervisor continued the program because “everyone liked it and assumed it would continue” (DAW Supervisor, personal communication, December 11, 2024). Dogs participating in the DAW program must be kept in the employee’s office. When an employee leaves their office during a workday, they may choose to leave their dog in their office. When leaving a dog in their office, the employee must utilize either a crate or gate to ensure safety of the dog and any employees entering the office. The DAW participant may use a leash to take their dog outside for basic care needs, such as walks and toileting. However, the dog should not be taken into the clinic or classrooms. This program is not intended for instances where a pet dog may be brought in for veterinary care or participation in a research activity. Instead, it is intended to honor the important role pets play in the lives of employees be providing an opportunity for their dogs to accompany them throughout the workday.

To be eligible for participation in DAW, an applicant must be employed by the veterinary college as staff, faculty, or a graduate student. The employee must have either an individual or a shared office. Students enrolled in degree programs in the veterinary college, but not employed by the college, are not eligible. The DAW applicant is expected to submit vaccine records to the DAW supervisor and then bring their dog in for a behavioral evaluation conducted by a board-certified veterinary behaviorist. The evaluation is a modified version of the American Kennel Club’s Canine Good Citizen Test, which examines behaviors such as interacting with strangers, interacting with other dogs, and reactions to distractions (American Kennel Club, n.d.). The examined dog can either pass, fail, or be deferred for future evaluation. Deferral results from unwanted behavior that has the potential for modification; deferred applicants are given recommendations to address the behavior and then invited to reapply. Unwanted behaviors include barking in the office, jumping on people, or pulling toward other dogs during walks. After passing the behavioral evaluation, the applicant is provided with a tag indicating their dog is approved for the DAW program. DAW dogs are expected to wear this tag on their collar anytime they are brought into the workplace. To protect the health of both DAW dogs and veterinary patients, all DAW participants are required to submit documentation of appropriate and up-to-date vaccinations and ensure that their dog is free of parasites. Additionally, DAW dogs are not allowed to enter areas of the veterinary school that present a high-risk of disease transmission.

Once approved, DAW participants must ensure their dogs continue to behave in accordance with their behavioral evaluation. If a DAW dog begins displaying unwanted behaviors, they can be reported to the DAW supervisor or school administrators. Dogs that were previously approved may be reevaluated and approval may be revoked if needed. These reevaluations provide the opportunity to objectively mediate interpersonal conflict and confirm the veracity of complaints. The DAW supervisor may also use the reevaluation as an opportunity to educate participants on dog care and dog needs, if the unwanted behavior is a result of human actions.

DAW participants in shared offices must obtain consent from all members of the office to bring their dog to work. Individuals at the veterinary school that do not have either a private or shared office, such as veterinary students, are not able to participate in the program. Additionally, certain buildings and areas within the veterinary school, such as laboratories and hospital areas, restrict access to only dogs receiving veterinary treatment or participating in educational activities. Staff, faculty, and graduate students in those areas, who would otherwise be approved to bring their DAW dog to work, are unable to participate in the program.

#### Recruitment

Participants were recruited from all staff, faculty, and graduate students within the veterinary school, regardless of their participation in the DAW program. At the time of recruitment, 40 dogs were registered to participate in DAW. The study team aimed to recruit 25 respondents with an approved DAW dog, and 25 respondents without an approved DAW dog.

An initial email was sent to faculty, staff, and graduate students within the veterinary school at the start of March 2024. This email contained a brief description of the study and a link to complete the online survey via Qualtrics Experience Management Software. Reminder emails were sent once weekly for the following 3 weeks. The survey was available for completion for approximately 1 month, with surveys closing at the end of March 2024.

This survey began with a question that presented a consent form and obtained informed consent. Additionally, the participant was informed that they would be eligible to receive $15 in the form of an Amazon e-gift card upon completion of the survey. At the end of the survey, respondents were asked if they would be interested in being contacted for an in-depth follow-up interview. Interview results were reported in Schieler et al. (2025). Recruitment efforts resulted in 88 total respondents, of which 64 (73%) had a dog and 24 (27%) did not have a dog. Of the 64 respondents with a dog, 23 (36%) had a dog registered in the DAW program and 41 (64%) had a dog that was not registered and therefore ineligible to come to the veterinary school campus as part of the DAW program.

### Measures

The online survey contained seven validated measures of workplace wellbeing, 11 open-ended qualitative short response questions, and 14 respondent characteristics questions pertaining to the respondent’s work and experiences with the DAW program. Due to the small size of the population being studied, typical demographics questions were not included to ensure responses were not identifiable. All qualitative short response questions and respondent characteristics questions were developed for this study. These questions are available in the Supplementary materials.

#### Respondent characteristics questions

Program-specific questions were aimed at understanding the respondent’s work role and experiences, as well as their status in relationship to the DAW program. Collected information included: (1) if they had a pet and what species it was, (2) if they participated in the DAW program, (3) their role at work and how many years they had been in that role, (4) their office type (e.g., shared, private, no office), (5) the amount of leave from work taken, and (6) the reason for taking leave from work.

#### Workplace strengths measures

Two measures were used to understand work-related strengths within the RPM framework. These measures focused on purpose, identity, and meaning in relation to work. This supported an investigation of the meaning-making strength in particular. The *Work-Related Quality of Life Scale* (WRQLS) (Edwards et al., 2009; Easton and Van Laar, 2018) is a validated self-report measure of the perceived quality of an individual’s work experience based on six sub scales: Job and Career Satisfaction (JCS), General Wellbeing (GWB), Stress at Work (SAW), Control at Work (CAW), Home-Work Interface (HWI), and Working Conditions (WCS). Responses to the WRQLS provided an overview of the respondent’s work experiences and perceptions. Scores for the sub scales were based on responses to 23 questions (e.g., “I often feel under pressure at work” and “I am satisfied with the training I receive in order to perform my present job”), and an additional question, “I am satisfied with the overall quality of my working life,” for use in measuring reliability and validity of the responses. Respondents answered on a five-point Likert scale ranging from 1 “Strongly Disagree” to 5 “Strongly Agree.” Scores were calculated for each sub scale by finding the average of the correlating questions for that scale, and then all sub scale scores were averaged for an overall work-related quality of life measure. The possible totals for subscale and overall scale responses ranged from 1 to 5, and higher scores indicated a more positive quality of work life.

The shortened version of the *Utrecht Work Engagement Scale* (UWES) (Schaufeli and Bakker, 2004) was used to understand the respondent’s levels of engagement with work. The UWES measured three aspects of work engagement: Vigor (e.g., “At my work, I feel bursting with energy”), Dedication (“I am enthusiastic about my job”), and Absorption (e.g., “I am immersed in my work”). Possible responses were on a scale ranging from 0 “Never” to 6 “Always – Every Day.” Responses were averaged for each sub scale, and those scores were then averaged to obtain the total work engagement score. The possible totals for subscale and overall scale responses ranged from 0 to 6, and higher scores indicated higher work engagement.

#### Workplace-related outcome measures

Measures of workplace-related outcomes were used to understand the associations between participating in a DAW program and workplace wellbeing within the RPM framework. These measures supported understanding of the regulatory strength, as the measured outcomes served as indicators of the respondent’s ability to self-regulate. To understand stress at work, the *Perceived Occupational Stress Scale* (POS) was included in the survey. The POS is a brief validated self-report measure of occupational stress over the previous 6 months, measured with four questions: “My work is stressful,” “Thinking about work makes me feel tense,” “At work I feel under pressure,” and “My work has negative effects on my health.” Response options were on a 5-point Likert scale ranging from 1 “Strongly Disagree” to 5 “Strongly Agree.” Responses to these four questions were averaged to compute the overall POS score, ranging from 1, the lowest level of perceived stress to 5, the highest level of perceived stress.

Feelings of burnout were measured via the *Maslach Burnout Inventory – General Survey* (MBI-GS) (Maslach et al., 1997), a 16 question validated measure with three sub scales: Exhaustion (e.g., “I feel emotionally drained from my work”), Cynicism (e.g., “I have become less enthusiastic about my work”), and Professional Efficacy (e.g., “I have accomplished many worthwhile things in this job”). Response options ranged from 0 “Never” to 6 “Every Day.” Responses were averaged for each sub scale to obtain individual scores for Cynicism, Exhaustion, and Professional Efficacy. The possible totals for each subscale ranged from 0 to 6. Higher scores on the Exhaustion and Cynicism scales and lower scores on the Professional Efficacy scale indicated higher burnout.

The shortened version of the *Turnover Intention Scale* (TIS-6) (Roodt, 2004) is a validated measure that consists of six questions examining the respondent’s intentions to stay with or leave an organization. Responses were on a 5-point Likert scale that varies by question, such as 1 “Never” to 5 “Always” for “How often have you considered leaving your job?” and 1 “Very satisfying” to 5 “Totally dissatisfying” for “How satisfying is your job in fulfilling your personal needs?” Response scores were summed for an overall score with a possible range of 6–30. A score above 18 indicated a desire to leave their organization and a score below 18 indicated a desire to stay with their organization.

#### General psychosocial outcome measures

Two psychosocial measures were used to further understand the associations between participating in a DAW program and workplace wellbeing. Both measures were validated short-form *Patient-Reported Outcomes Measurement Information System* (PROMIS) (Cella et al., 2019) self-report measures. These measures were used to capture perceptions of mental health over the last 7 days: PROMIS Anxiety and PROMIS Depression. Both measures consisted of four questions (e.g., Anxiety: “My worries overwhelmed me”; Depression: “I felt worthless”) with a 5-point Likert scale for responses, which ranged from 1 “Never” to 5 “Always.” Responses were summed to provide an overall raw summed score for each measure. Raw scores were then converted into T-scores using the accompanying conversion table. The possible range for PROMIS anxiety scores was 40.3 to 81.6. The possible range for PROMIS depression scores was 41.0 to 79.4. Higher scores indicated higher anxiety or depression, respectively.

#### Qualitative questions

The survey contained 11 open response questions. If the respondent indicated that they participated in the DAW program, they were asked to describe: (1) their motivation for bringing their dog to work, (2) their motivation for how often they brought their dog to work, (3) how their dog interacted with others (human or animal) while at work, (4) their overall experience with the DAW program, and (5) any suggestions for changing the program. If the respondent did not participate in the DAW program but still had a dog they brought to the workplace, they were asked to describe: (1) what determined whether they brought their dog in or not, and (2) why they had not registered their dog in the DAW program. If respondents indicated they did not bring their dog into the workplace or did not have pets, they were asked to describe: (1) their experience with the DAW program, (2) what they would change about the program, and (3) their overall thoughts on the program. Finally, all respondents were asked, “Is there anything else that you would like to share with the research team?” Open-ended questions allowed for further insight into the meaning-making strength, as well as an understanding of the other three strengths. Taken together, these qualitative and quantitative measures may support an understanding of the Resilience Portfolios and wellbeing of employees at a veterinary college with a long-standing DAW program.

### Data analysis

Respondent characteristics were descriptively summarized and compared via a Fisher’s exact test. This test was chosen over a chi-squared test due to the small sample size (Campbell, 2007). Computed scores for each workplace wellbeing measure were compared between participants of the DAW program and the control group (all respondents not participating in the program) via Welch’s t-tests. Using Welch’s t-test allowed for the possibility of unequal standard deviations and accounted for the difference between the DAW and control group sample sizes (West, 2021). In light of the exploratory nature of the study, after data collection additional post-hoc t-tests were performed. These post-hoc tests were used to compare means between unique subsets identified within the respondents, specifically: DAW participants compared to respondents with no pets, respondents with any dog and respondents with no dog, and respondents with any pet and respondents with no pet. In line with recommendations in published literature, Bonferroni corrections were not applied because the t-test comparisons were considered as individual results and not as a single universal null hypothesis; and because the post-hoc tests were exploratory comparisons intended to guide future investigations (Perneger, 1998; Armstrong, 2014). It is important to note that this approach reduced the chance of Type II error but increased the chance of Type I error for these post-hoc analyses (Rothman, 1990). Post-hoc analyses were not intended to provide direct conclusions about the effects of pets on workplace wellbeing. All analyses were completed using R version 4.4.1 (R Core Team, 2024) with a significance level of *p* < 0.05.

Qualitative responses to open-ended questions were coded via an inductive content analysis method using NVivo 15 software to understand the respondent’s experiences with the DAW program and reasons for participating or not participating (Elo and Kyngäs, 2008). All responses were considered together, regardless of DAW participation status. First, responses to each open-ended question were read through multiple times to obtain a sense of reoccurring phrases and words (Hsieh and Shannon, 2005). After gaining familiarity with responses and reoccurring phrases, open coding was used to note emerging categories and develop descriptions of these categories (Downe-Wamboldt, 1992). After initial review of the responses, investigator triangulation was employed to reduce bias while preserving the richness of varied respondent viewpoints (Archibald, 2016). Specifically, two members of the research team reviewed and approved the categories. Then, the responses were read through again to further refine categories and allow for creation of broader themes for the previously identified categories (Elo and Kyngäs, 2008). These themes and sub-themes were triangulated via collaborative review by the same two members of the research team. Any disagreements were discussed and categories were edited until a consensus was reached. The qualitative analysis methodology employed in this study aligned with the qualitative approach to establishing intercoder reliability outlined by Cofie et al. (2022).

## Results

### Respondent characteristics

Due to the small population that the sample was drawn from, standard measures of respondent demographics were not included in the survey to ensure confidentiality. Instead, other respondent characteristics specific to the DAW program were measured. These program-specific questions pertained to the respondent’s work, pets, and DAW participation status.

A total of 88 survey responses were included in the data analysis. Table 1 displays respondents grouped into DAW program participants (*n* = 23) or control (*n* = 65); the control group consisted of respondents that had a dog but did not participate in DAW (*n* = 41), and respondents without a dog (*n* = 24). An additional subset of the control group, respondents with no pets of any species (*n* = 13), was also identified for use in post-hoc analyses. Missing data are included in the table as “No response.”

**Table 1:** Respondent workplace characteristics and *p*-value for Fisher’s exact test for DAW participants (*n* = 23) and the control group (*n* = 65).

Characteristic	Registered DAW	Control	Overall	*p*-value
Has dog, *n* (%)				<0.001
Dog	23 (100%)	41 (63%)	64 (73%)	
No dog	0 (0%)	24 (37%)	24 (27%)	
Has any pet, *n* (%)				0.018
Pet	23 (100%)	52 (80%)	75 (85%)	
No pet	0 (0%)	13 (20%)	13 (15%)	
Position, *n* (%)				0.111
Staff	15 (65%)	41 (64%)	56 (64%)	
Faculty	8 (35%)	12 (19%)	20 (23%)	
Graduate student staff	0 (0%)	9 (14%)	9 (10%)	
Postdoc staff	0 (0%)	2 (3.1%)	2 (2.3%)	
Other	0 (0%)	0 (0%)	0 (0%)	
No response	0	1	1	
Clinical role, *n* (%)				0.333
Yes	12 (52%)	25 (40%)	37 (43%)	
No	11 (48%)	38 (60%)	49 (57%)	
No response	0	2	2	
Office type, *n* (%)				0.215
Shared office	12 (52%)	36 (58%)	48 (56%)	
Private office	11 (48%)	20 (32%)	31 (36%)	
I do not have an office	0 (0%)	6 (9.7%)	6 (7.1%)	
No response	0	3	3	
Years in Role, *M* (*SD*)	6.96 (5.76)	5.02 (7.23)	5.54 (6.88)	0.031
No response	0	3	3	
Number of days working in the hospital, *M* (*SD*)	4.42 (1.38)	4.42 (1.28)	4.42 (1.30)	0.846
No response	11	41	52	
Number of days of sick leave taken for personal reasons, *M* (*SD*)	7.48 (6.78)	10.18 (10.96)	9.45 (10.03)	0.462
No response	0	3	3	
Number of days of sick leave taken for family reasons, *M* (*SD*)	1.96 (2.67)	4.26 (7.64)	3.64 (6.73)	0.397
No response	0	3	3	
Number of days of sick leave taken for pet-related reasons, *M* (*SD*)	0.35 (0.71)	0.50 (1.02)	0.46 (0.95)	0.852
No response	0	3	3	
Number of days taken off work due to pet care responsibilities, *M* (*SD*)	0.04 (0.21)	0.29 (0.76)	0.22 (0.65)	0.118
No response	0	14	14	

The Overall column represents the total number of respondents that selected the given answer, and their percentage out of the total sample (*N* = 88). Significance was set at *p* < 0.05.

In Table 1, the following categories provided information on who does or does not participate in the DAW program and who is or is not eligible to participate: Has Dog, Position, Clinical Role, Office Type. Survey respondents were required to be employed at least part-time by the veterinary school, in alignment with participation criteria for the DAW program (represented by Position responses in Table 1). Under half of all respondents had roles that involved clinical duties (represented by Clinical Role in Table 1), in which staff work with patients at the college’s public-facing veterinary hospitals. DAW participants are required to have an office that can be closed with a door, either shared with one or more other employees, or private to just them (Office Type in Table 1). Respondents that did not have an office were ineligible to participate in the DAW program.

### Quantitative results

Seven instruments were used to quantitatively assess workplace wellbeing. The WRQLS showed good internal consistency overall (23 items; *α* = 0.84), as well as acceptable to good internal consistency across all six subscales: JCS (6 items; *α* = 0.76), GWB (6 items; *α* = 0.86), SAW (2 items; *α* = 0.73), CAW (3 items; *α* = 0.79), HWI (3 items; *α* = 0.85), WCS (3 items; *α* = 0.74). The UWES showed strong internal consistency overall (9 items; *α* = 0.90), while the internal consistency of the subscales varied: Vigor (3 items; *α* = 0.87), Dedication (3 items; *α* = 0.86), Absorption (3 items; *α* = 0.67). The POS showed good internal consistency (4 items; *α* = 0.86). The MBI showed acceptable internal consistency (16 items; *α* = 0.76) and the subscales showed acceptable to good internal consistency: Exhaustion (5 items, *α* = 0.89), Cynicism (5 items, *α* = 0.77), Professional Efficacy (6 items, *α* = 0.78). The TIS showed good internal consistency (6 items, *α* = 0.80). The PROMIS anxiety scale showed good internal consistency (4 items, *α* = 0.82) and the PROMIS depression scale showed acceptable internal consistency (4 items, *α* = 0.78).

We compared each wellbeing measure between DAW participants and the control group using Welch’s t-tests, as shown in Table 2. All wellbeing measure means were in line with average population values for the total sample, as well as for both DAW participants and control respondents. None of the measures of wellbeing differed significantly between groups. However, workplace vigor, defined as “high levels of energy and resilience, the willingness to invest effort, not being easily fatigued, and persistence in the face of difficulties,” approached significance at *p* = 0.058 (Schaufeli et al., 2006). DAW participants showed higher average vigor scores (3.94) compared to the control group (3.35), indicating that DAW participants reported higher levels of vigor in the workplace. Both groups had an average turnover intention score under 18 (DAW = 16.14, control = 15.07), indicating a desire to stay at their current workplace.

**Table 2:** Wellbeing measure means and Welch’s t-test results comparing means for DAW participants (*n* = 23) and the control group (*n* = 65).

Validated wellbeing measure	Registered DAW	Control	Overall			
	*M*	*M*	*M*	*t* score	*df*	*p*-value
Work-Related Quality Of Life Scale (WRQLS)						
WRQLS: General Well Being	3.72	3.51	3.57	1.50	46.04	0.141
WRQLS: Home-Work Interface	3.71	3.70	3.70	0.06	54.40	0.955
WRQLS: Job Career Satisfaction	3.94	3.84	3.87	0.73	40.01	0.469
WRQLS: Control at Work	3.58	3.41	3.45	0.84	40.42	0.408
WRQLS: Working Conditions	3.96	3.8	3.84	1.21	54.67	0.232
WRQLS: Stress at Work	2.55	2.83	2.75	−1.15	31.96	0.26
WRQLS: Overall	3.57	3.51	3.53	0.48	46.37	0.633
Utrecht Work Engagement Scale (UWES)						
UWES: Vigor	3.94	3.35	3.52	1.96	33.80	0.058
UWES: Dedication	4.36	4.4	4.39	−0.12	32.31	0.907
UWES: Absorption	4.12	3.97	4.01	0.58	36.09	0.563
UWES: Overall	4.14	3.91	3.97	0.90	32.53	0.372
Turnover intention scale (TIS)						
TIS	16.14	15.07	15.37	0.95	42.53	0.349
Patient-Reported Outcomes Measurement Information System (PROMIS)						
PROMIS: Anxiety Total	50.24	51.92	51.45	−0.77	38.74	0.447
PROMIS: Depression Total	49.7	50.85	50.52	−0.64	43.84	0.527
Perceived Occupational Stress (POS)						
POS	3.02	2.62	2.73	1.69	39.70	0.098
Maslach Burnout Inventory (MBI)						
MBI: Emotional Exhaustion	2.91	2.64	2.71	0.74	35.94	0.463
MBI: Cynicism	2.22	1.79	1.91	1.59	42.59	0.118
MBI: Professional Efficacy	4.71	4.57	4.61	0.62	35.23	0.538

Significance was set at *p* < 0.05.

While the control group was homogenous regarding DAW participation, various other companion animal subsets were present (any pet, dog not registered in DAW, pets other than dog, no pets). Therefore, three additional post-hoc analyses were performed to further explore impacts to workplace wellbeing. Comparing respondents with pets and respondents with no pets (Table 3) showed significant differences on three measures: WRQLS general wellbeing (*p* = 0.022), PROMIS anxiety (*p* = 0.016), and PROMIS depression (*p* = 0.006). Respondents with any dog compared to respondents with no dog (Table 4) showed significant differences on two measures: PROMIS anxiety (*p* = 0.04) and PROMIS depression (*p* = 0.035). Anxiety was significantly lower for respondents with any dog (*M* = 50.42) compared to respondents with no dog (*M* = 54.88). Depression was also significantly lower for respondents with any dog (*M* = 49.51) compared to respondents with no dog (*M* = 53.91). Aligned with pets compared to no pets, comparing DAW participants to respondents with no pets (Table 5) showed significant differences on three measures: WRQLS general wellbeing (*p* = 0.022), PROMIS anxiety (*p* = 0.016), and PROMIS depression (*p* = 0.006). General workplace wellbeing was significantly higher for DAW participants (*M* = 3.72) than respondents without pets (*M* = 3.09). Anxiety was significantly lower for DAW participants (*M* = 50.24) compared to respondents without pets (*M* = 56.69). Depression was also significantly lower for DAW participants (*M* = 49.7) compared to respondents without pets (*M* = 56.51).

**Table 3:** Post-hoc Welch’s t-tests comparing means of wellbeing measure scores for people with any kind of pet (*n* = 75) compared to people with no pets (*n* = 13).

Validated wellbeing measure	Any pet	No pet	Overall			
	*M*	*M*	*M*	*t* score	*df*	*p*-value
Work-Related Quality of Life Scale (WRQLS)						
WRQLS: General Well Being	3.63	3.09	3.57	2.38	9.82	0.039
WRQLS: Home-Work Interface	3.77	3.22	3.70	1.35	9.13	0.209
WRQLS: Job Career Satisfaction	3.89	3.72	3.87	1.01	11.36	0.334
WRQLS: Control at Work	3.50	3.07	3.45	1.46	10.07	0.174
WRQLS: Working Conditions	3.88	3.56	3.84	1.35	9.55	0.209
WRQLS: Stress at Work	2.76	2.67	2.75	0.22	9.02	0.828
WRQLS: Overall	3.57	3.22	3.53	1.65	9.62	0.132
Utrecht Work Engagement Scale (UWES)						
UWES: Vigor	3.55	3.26	3.52	0.76	10.52	0.465
UWES: Dedication	4.39	4.41	4.39	−0.50	9.67	0.962
UWES: Absorption	4	4.15	4.01	−0.46	10.58	0.655
UWES: Overall	3.98	3.94	3.97	0.13	10.78	0.901
Turnover Intention Scale (TIS)						
TIS	15.10	17.44	15.37	−1.50	10.55	0.162
Patient-Reported Outcomes Measurement Information System (PROMIS)						
PROMIS: Anxiety Total	50.77	56.69	51.45	−3.00	15.63	0.009
PROMIS: Depression Total	49.74	56.51	50.52	−3.60	13.41	0.003
Perceived Occupational Stress (POS)						
POS	2.68	3.14	2.73	−1.25	9.85	0.239
Maslach Burnout Inventory (MBI)						
MBI: Emotional Exhaustion	2.63	3.36	2.71	−1.31	9.63	0.221
MBI: Cynicism	1.95	1.64	1.91	0.67	9.64	0.519
MBI: Professional Efficacy	4.63	4.48	4.61	0.43	9.83	0.675

Significance was set at *p* < 0.05.

**Table 4:** Post-hoc Welch’s t-tests comparing means of wellbeing measure scores for people with a dog, regardless of DAW status (*n* = 64) compared to people with no dog (*n* = 24).

Validated wellbeing measure	Any dog	No dog	Overall			
	*M*	*M*	*M*	*t* score	*df*	*p*-value
Work-Related Quality of Life Scale (WRQLS)						
WRQLS: General Well Being	3.64	3.32	3.57	1.79	25.08	0.086
WRQLS: Home-Work Interface	3.74	3.57	3.70	0.65	25.62	0.52
WRQLS: Job Career Satisfaction	3.88	3.82	3.87	0.40	28.70	0.69
WRQLS: Control at Work	3.46	3.43	3.45	0.17	28.45	0.868
WRQLS: Working Conditions	3.88	3.70	3.84	0.95	23.17	0.35
WRQLS: Stress at Work	2.75	2.75	2.75	0	26.44	1
WRQLS: Overall	3.56	3.43	3.53	0.83	26.42	0.413
Utrecht Work Engagement Scale (UWES)						
UWES: Vigor	3.56	3.37	3.52	0.65	30.38	0.518
UWES: Dedication	4.36	4.48	4.39	−0.41	27.75	0.682
UWES: Absorption	4	4.06	4.01	−0.23	33.19	0.82
UWES: Overall	3.97	3.97	3.97	0.02	33.09	0.983
Turnover Intention Scale (TIS)						
TIS	15.38	15.33	15.37	0.04	28.09	0.969
Patient-Reported Outcomes Measurement Information System (PROMIS)						
PROMIS: Anxiety Total	50.42	54.88	51.45	−2.14	33.02	0.04
PROMIS: Depression Total	49.51	53.91	50.52	−2.21	27.94	0.035
Perceived Occupational Stress (POS)						
POS	2.73	2.76	2.73	−0.15	28.49	0.882
Maslach Burnout Inventory (MBI)						
MBI: Emotional Exhaustion	2.67	2.84	2.71	−0.45	28.28	0.655
MBI: Cynicism	2.02	1.56	1.91	1.43	25.38	0.166
MBI: Professional Efficacy	4.61	4.62	4.61	−0.07	30.18	0.948

Significance was set at *p* < 0.05.

**Table 5:** Post-hoc Welch’s t-tests comparing means of wellbeing measure scores for DAW participants (*n* = 23) compared to people with no pets (*n* = 13).

Validated wellbeing measure	Registered DAW	No pets	Overall			
	*M*	*M*	*M*	*t* score	*df*	*p*-value
Work-Related Quality of Life Scale (WRQLS)						
WRQLS: General Well Being	3.72	3.09	3.57	2.60	12.69	0.022
WRQLS: Home-Work Interface	3.71	3.22	3.70	1.18	10.34	0.265
WRQLS: Job Career Satisfaction	3.94	3.72	3.87	1.16	17.34	0.26
WRQLS: Control at Work	3.58	3.07	3.45	1.55	14.20	0.143
WRQLS: Working Conditions	3.96	3.56	3.84	1.61	10.95	0.137
WRQLS: Stress at Work	2.55	2.67	2.75	−0.26	12.93	0.798
WRQLS: Overall	3.57	3.22	3.53	1.57	12.11	0.143
Utrecht Work Engagement Scale (UWES)						
UWES: Vigor	3.94	3.26	3.52	1.53	17.07	0.145
UWES: Dedication	4.36	4.41	4.39	−0.09	15.35	0.929
UWES: Absorption	4.12	4.15	4.01	−0.07	16.74	0.945
UWES: Overall	4.14	3.94	3.97	0.55	18.78	0.587
Turnover Intention Scale (TIS)						
TIS	16.14	17.44	15.37	−0.76	14.81	0.459
Patient-Reported Outcomes Measurement Information System (PROMIS)						
PROMIS: Anxiety Total	50.24	56.69	51.45	−2.59	25.28	0.016
PROMIS: Depression Total	49.7	56.51	50.52	−3.09	20.65	0.006
Perceived Occupational Stress (POS)						
POS	3.02	3.14	2.73	−0.29	13.61	0.776
Maslach Burnout Inventory (MBI)						
MBI: Emotional Exhaustion	2.91	3.36	2.71	−0.72	14.14	0.482
MBI: Cynicism	2.22	1.64	1.91	1.19	12.48	0.255
MBI: Professional Efficacy	4.71	4.48	4.61	0.61	14.81	0.548

Significance was set at *p* < 0.05.

### Qualitative results

Qualitative analysis of open-ended survey responses revealed three themes consistent with the literature on the benefits of human-animal interactions: (1) Mental Health, (2) Physical Health, and (3) Social Health. A fourth theme, Logistics, emerged related to remarks pertaining to participation barriers and practical program implications. Three RPM strengths (regulatory, interpersonal, and environmental) aligned with the qualitative findings.

#### Theme one: mental health

Respondents mentioned mental health in three contexts: mental health improvements due to the DAW program (for both humans and dogs), dog fearfulness when brought to work, and negative mental health impacts due to work.

Sub-theme one: mental health improvements. Mental health support and improvements were perceived for both DAW dogs, and for humans regardless of program participation, through mentions of increased happiness, lowered stress, and direct references to mental health and wellbeing. One response described interactions with dogs as “puppy therapy.” Regardless of if the interaction was with their own dog or with someone else’s dog, multiple respondents stated that interacting with DAW dogs made their day better. Multiple respondents perceived lowered stress from the presence of dogs. One DAW participant shared, “it’s really nice to have your pet with you, especially when you have a stressful week.” They continued on to add, “He’s always happy to be here!” This two-way direction of perceived support and perceived improvements to mental health was mentioned in multiple responses, with one DAW participant noting, “My dog in my office helps me because I see how happy she is to see me at the end of the day.”

Sub-theme two: dog fearfulness and work. Dog fearfulness was noted by some respondents in the workplace, while some other dogs were explicitly brought to work to alleviate fear of bad weather. Only one respondent said that this fearfulness prevented them from bringing their dog to work: “Unfortunately, my pup gets very stressed at the hospital even just hanging out in my office.” Other displays of fearfulness were in response to particular stimuli, such as “[human] males that she meets for the first time” or “jingling keys.”

Sub-theme three: mental health impacts from work. When respondents spoke about the cause of their stress and mental health challenges, they explicitly referenced their work environment, expectations of their role, or the veterinary field generally. In line with research showing high levels of burnout in the veterinary field (Takefuji, 2025), one respondent shared:

I think veterinary medicine in general, despite what department you are in has a high burn out due to the high emotional compassion we exert with our patients every day. We hold such a high responsibility to care for people’s pets and make sure that they get the highest quality of care when that sometimes means not looking out for our own mental health and wellbeing.

Another respondent described positive feelings toward their personal work experience, sharing, “I enjoy my job and feel that I contribute a lot to the college” before going on to share their concern about their coworkers overall by saying, “I think the [vet college] family is stretched thin and almost everyone has a full plate.” They felt this “takes away from people feeling good about what they contribute and accomplish.” Echoing this, another respondent shared that one of the main causes of work-related stress for them was “not feeling like I am getting help/support I need to perform daily work/tasks and not feeling appreciated.”

#### Theme two: physical health

Every mention of physical health by respondents indicated perceptions that the DAW program provided increased support for physical health. Specific sub-themes that emerged in relation to physical health included perceived increases in opportunities for exercise and movement, as well as the ability to manage daily pet care and veterinary care needs.

Sub-theme one: increased opportunities for exercise. Interacting with dogs at work was perceived to increase the opportunities for exercise for both dogs and humans participating in the DAW program. Respondents shared that DAW dogs had the opportunity to play with others and had more opportunities to go for walks or be taken outside during the day. One participant noted that they mainly brought their dogs to work when “staying late for some reason so I can walk them,” providing opportunities for both the respondent and their dog to go on walks they otherwise may not have been able to due to long work hours.

Sub-theme two: daily pet care and veterinary care. The ability to easily address daily, basic pet care needs in a timely manner was a common reason that many respondents participated in the DAW program. Some respondents did not “have anyone else to reliably let [my dog] out.” Others described “extra-long day[s]” at work that prevented them from providing timely pet care. One respondent was concerned with “how long [my dogs] will be without a potty break” if they are not brought to their workplace. The ability to access veterinary care for their dogs was also a common reason for respondents to bring their dogs to work. One respondent said they would bring their dog in to “be seen for illness…or come in for wellness appointments,” and another brought their dog in for “rehab appointments.” Even if they were not attending veterinary appointments, having their dog at work through the DAW program allowed respondents to monitor illnesses and issues. One respondent shared that they bring their dog in if they “need to watch her for health concerns.”

#### Theme three: social health and social interactions

Responses regarding social health indicated that social interactions related to the DAW program were generally perceived as positive. Respondents also perceived increases in opportunities for interaction between themselves and their dog, between themselves and their coworkers or students, between their dog and other dogs, and between their dog and their coworkers or students. Two distinct sub-themes for social health and social interactions emerged; these were perceived support for the already existing respondent-dog relationships, and perceived increases in opportunities for positive human-human, human-dog, and dog-dog social interactions.

Sub-theme one: support for respondent-dog relationships. Many respondents expressed worry about their dog being alone at home for extended periods of time as a reason for participating in the DAW program. This was particularly prevalent for respondents who felt they worked long hours. One respondent said, “If I have a long workday, I don’t want to leave her home for so long” and another stated, “If I’m here for longer than 8 hours I bring her.” The DAW program was also used by some respondents as support for their overall relationship with their dog, as evidenced by one DAW participant sharing, “she is very attached to me so I take her everywhere with me.” One respondent shared, “I would not be able to have a dog if I could not…bring my dog to work with me every day.”

Sub-theme two: positive or increased social interactions. Respondents felt that the DAW program supported social interactions for staff and faculty at the veterinary college by “bring[ing] people together.” Coworkers that brought their DAW dogs into work were seen as “engaging when asked about their dog.” These perceived positive interpersonal effects were even seen to spill over to home life, with one respondent saying, “It [the DAW program] has been great – very beneficial to my family.” Interactions between humans and DAW dogs were consistently seen by respondents as positive experiences. DAW dogs were described as enjoying “greeting colleagues and students” and being friendly and “very people oriented.” DAW participants generally felt that their dogs were happy to interact with their coworkers, with one respondent saying “[my dog] loves everyone and everything.” Respondents also felt that participation in the DAW program also provided DAW dogs with opportunities to socialize with new humans and dogs.

#### Theme four: logistics

The final theme represented in survey responses was logistics, which encompassed practical implications for employers running DAW programs or looking to implement DAW programs in the future. The three sub-themes identified are barriers to participation, program perceptions, and DAW program recommendations.

Sub-theme one: barriers to participation. Lack of awareness about the DAW program was commonly given as a reason for not participating. Some respondents “didn’t know there was such a thing” while others were aware of the program but “don’t know how to register.” Other respondents identified scheduling and time demands as their primary reason for not participating, with some specifically mentioning their clinical requirements as veterinary professionals. Additionally, interactions with others caused some respondents to avoid bringing their DAW dog to the workplace. Some respondents struggled with “others who bring their [non-DAW] dogs in,” indicating inter-dog conflict. Others expressed concerns about being respectful in shared office spaces: “I do think it is different in solo office vs a shared office space in regards to if all are on board or if several in same office bring dogs on same days.” Office spaces also limited participation based on their physical location. Some buildings within the veterinary college were identified as not allowing dogs, even if they were DAW approved. This created a barrier for respondents who may have otherwise been interested in participating, based on their assigned office space. One respondent suggested that it should be allowed for all “dogs that have been approved as [DAW] dogs to be in the shared offices with other coworker and faculty approval,” addressing both concerns of physical location and shared office spaces.

Sub-theme two: program perceptions. A few respondents indicated that their “only interaction [with DAW staff] has been to get [my dog] enrolled in the program” and no further assessments or check-ins were performed. While some did not see this as an issue, a few respondents mentioned that it meant “a lot of people sometimes bring their bad dogs.” One respondent noted that they have encountered dogs in the workplace that have “barked or charged the closed see-through gate.” On the other hand, many respondents expressed very positive perceptions of the program: “I think this is a great initiative and program!” During the registration process, staff were seen as “very kind and helpful” and “there was no hassle when requesting the evaluation.” Respondents were appreciative of the ability to bring their dog to work with them, feeling “grateful that this is an option.”

Sub-theme three: DAW program recommendations. Respondents provided practical suggestions to further enhance the benefits they perceived from the DAW program. Annual recertification was most commonly suggested as an improvement, as “behaviors can change over time.” Respondents understood that this may be difficult to implement with limited staff: “Having only one [staff member] that could certify a dog could be overwhelming at times.” Respondents also indicated that it was important that “everyone that brings there [*sic*] dogs in should be in the program.” One suggestion to address this was “doing random checks if people’s dogs are [DAW] dogs” because “you can’t tell who has been approved to be here” despite the DAW program requiring approved dogs to wear a DAW tag. Other options to support employee wellbeing were shared by a few respondents. Both flexible hours and remote hours were suggested as supplemental options that “would benefit many staff members mental health,” either in combination with or as an alternative to the DAW program.

## Discussion

This study examined perceptions of and experiences with a DAW program through the lens of the RPM (Grych et al., 2015; Banyard et al., 2025). Within the RPM framework, the staff at this veterinary college appear to have sufficient strengths to mitigate some of the high-stress nature of the veterinary field, regardless of DAW participation status. In particular, workplace-related meaning-making strengths such as identity, purpose, engagement, and satisfaction are highlighted by the WRQLS and WES results. Dogs have previously been shown to support identity formation and feelings of purpose, so it is possible that they are enhancing meaning-making in this sample across groups (Brooks et al., 2018). Results from the WRQLS and WES did not differ between DAW program participants and non-participants. This indicated that staff at this veterinary college may have similar strengths to draw from, regardless of whether or not they bring a dog to work. In line with this finding, neither group differed on workplace or psychosocial outcome measures. Notably, both groups exhibited workplace and mental health outcomes better than those typically seen in the veterinary field, indicating that this group may have regulatory strengths, regardless of DAW participation status (Takefuji, 2025). When participants were divided into subsets based on pet status, reported mental health and wellbeing were consistently better for respondents with pets, and for respondents with dogs in particular. This may indicate a connection between workplace wellbeing and companion animals that warrants further investigation.

Qualitative findings revealed particular strengths that respondents drew from to mitigate stress and support resilience and wellbeing. DAW participants perceived that bringing a dog to work allowed them to engage in physical activity more often than they otherwise would, because dogs require regular periods of time outside. This physical activity likely served as an immediate regulatory strength for DAW participants by providing a meaningful break and increasing mental and cognitive wellbeing (Radwan et al., 2022; Whittenbury et al., 2025). Additionally, the long-term health benefits of consistent exercise can also serve as a regulatory strength (Radwan et al., 2022; Whittenbury et al., 2025). Another regulatory strength available to all veterinary staff at this school is the ability to physically interact with the dogs being brought to work. Petting animals has been shown to buffer stress responses in multiple settings; in the workplace, employees in high-stress jobs often choose petting animals as a relaxation activity when available (Applebaum et al., 2021; Whittenbury et al., 2025). The ability to pet their own or their coworker’s dog after a stressful experience may serve as an immediate regulatory strength for staff at this veterinary school.

Healthy relationships with family members serve as an interpersonal strength (Banyard et al., 2025; Whittenbury et al., 2025). Dogs are often considered a family member in households in the United States (Volsche, 2019). Therefore, the DAW program may support and strengthen the relationship between the participating staff member and their dog, which in turn could enhance the interpersonal strengths they draw on to maintain wellbeing in a high-stress environment. Multiple participants emphasize that this program improved their ability to care for their dog’s needs. Veterinary work often involves long hours away from home. Participants indicated that being able to bring their dog to work meant that participants could take their dog for walks, play with them, and provide bathroom breaks throughout the day, and not just before and after work. Feelings of dog-related guilt, stemming from perceiving themselves as unable to care for their dog properly, have been shown to damage human-animal relationships and negatively affect both work and personal life (Kogan et al., 2022, 2023; Junça-Silva, 2022). By allowing employees to bring their dog in, employers may be relieving this guilt and enhancing relationships between employees and their dogs. Additionally, the ability to address their dog’s needs throughout the day may improve dog welfare. Notably, multiple participants felt their dogs were happy to come to the workplace, and that they enjoyed meeting new people and other dogs. The potential for bidirectional benefits, improving wellbeing for employees and their dogs, may set DAW programs apart from other workplace approaches for improving wellbeing.

Within the workplace, positive social interactions with colleagues and managers have been shown to improve resilience and wellbeing in high-stress environments (Whittenbury et al., 2025). On the other hand, if work relationships are strained, as often happens in high-stress work environments, wellbeing has been shown to be negatively impacted (Whittenbury et al., 2025). Our findings indicated that participants perceived that both the amount of social interactions increased and that social interactions were generally perceived as more positive. This was true even for those who did not bring a dog to work, and the improvements were even seen to carry over to positive interactions with students. The DAW program appears to be perceived as generally bolstering and improving social relationships in this workplace, despite the high stress of the veterinary setting.

Whittenbury et al. (2025) found environmental strengths to be particularly impactful on resilience for employees in high-stress careers. In the workplace, environmental strengths depend on intentional design and positive maintenance of the workplace. This can include supportive workplace policies, a safe working environment, and perceptions that one’s employer values them (Banyard et al., 2025; Whittenbury et al., 2025). In the context of the DAW program, respondents felt thankful that they were able to bring their dog to work and appreciated the work that their coworkers and employer put into maintaining the program. Research has shown that employees perceive benefits from dogs in the workplace, even when they are not participating in the program (Wells and Perrine, 2001; Wagner and Pina E Cunha, 2021; Sousa et al., 2022). Staff at this veterinary school may perceive the DAW program as a supportive workplace policy, and therefore an environmental strength, even if they do not participate. The perceived benefits to welfare for participating DAW dogs may further support the perception of a supportive workplace. Productivity, work engagement, and wellbeing improve when employees feel that their employer shares their own values, and employees may feel more secure in their work environment (Junça-Silva and Galrito, 2024). One of the most commonly given reasons for entering the veterinary medicine field is to support animal health and improve animal welfare (Daly and Erickson, 2012). Within a veterinary college, implementing a DAW program with perceived benefits to animal welfare may result in perceived value alignment between employee and employer. The long-standing nature of this program may be perceived as a long-standing commitment to this value, which may further solidify the perceived alignment. In line with this, access to the DAW program at this veterinary college may be motivating respondents to stay in their current role, as indicated by the turnover intention measure and the average tenure of respondents. When polled, employees in the veterinary field at large consistently indicated that they intended to reduce their working hours or stop working in the veterinary field altogether within 5 years (Heath, 1998; Kogan and Rishniw, 2024). In contrast, our study found that average turnover intentions were below the threshold for intention to leave, and respondent tenures, on average, exceeded the five-year cut-off point. The DAW program may be viewed as a unique and valuable resource that is not commonly found at other employers.

While meaning-making strengths were shown in quantitative findings, they did not stand out in the qualitative findings. This is likely due to the nature and focus of the short answer questions utilized in this survey, as support of meaning and purpose were identified in the semi-structured interviews reported in Schieler et al. (2025).

### Mixed methods integration

**Figure 1:**
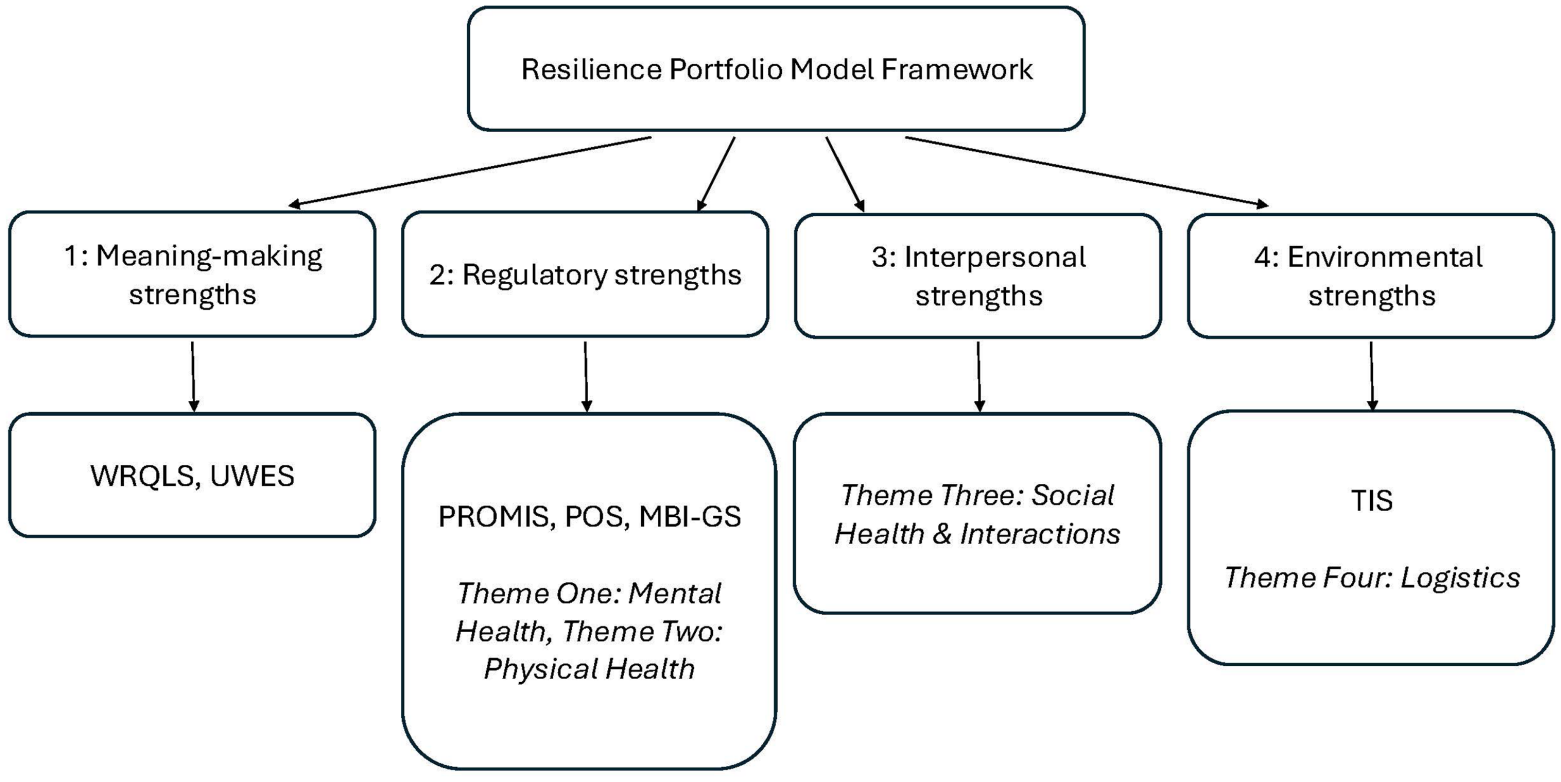
Integration of RPM framework and results. Quantitative measures are in plain text, qualitative themes are in *italics*. Includes the Work-Related Quality of Life Scale (WRQLS), Utrecht Work Engagement Scale (UWES), Patient-Reported Outcomes Measurement Information System (PROMIS), Perceived Occupational Stress Scale (POS), Maslach Burnout Inventory-General Survey (MBI-GS), & Turnover Intention Scale (TIS).

Taken together, the quantitative and qualitative findings indicate that the employees at this veterinary school, regardless of DAW program participation, may possess multiple strengths that support their wellbeing and resilience despite the high-stress environment. Figure 1 summarizes the relationship between the RPM framework, quantitative measures, and qualitative themes. Quantitative results suggest that staff of this veterinary hospital may have meaning-making and regulatory strengths. Qualitative results suggest that staff possess regulatory, interpersonal, and environmental strengths. However, there is disagreement between qualitative and quantitative results. Specifically, quantitative results demonstrate strengths regardless of DAW participation status, while the benefits of bringing their own dog to work was consistently highlighted in qualitative results. It is necessary to integrate these findings by further exploring the overlaps and disagreements between qualitative and quantitative results. While qualitative results suggested benefits to workplace wellbeing due to DAW participation, quantitative results did not. Some other studies of wellbeing and human-animal interactions in different settings or looking at different species have also found disagreement between qualitative and quantitative measures (Hoffman et al., 2018; Smith et al., 2018; Gee et al., 2019; Clements et al., 2024). In those studies, as with the current study, quantitative findings were generally null, while qualitative responses indicated perceptions of improvement or positive animal effects.

One possible explanation for this is that the quantitative self-report measures in this study may not measure the same concepts discussed in qualitative responses. Stress was specifically mentioned repeatedly in open-ended responses. While the POS and the WRQLS Stress at Work subscale both measured stress, they focused specifically on stress at work. It is possible that incorporating a more generalized stress measure would capture the stress mentioned in qualitative responses. Additionally, using a measure aimed at capturing human-animal interaction effects, such as the Guilt about Dog Parenting Scale (Kogan et al., 2022) or Human-Animal Interaction at Work Scale (Junça-Silva, 2024), may have captured perceptions expressed qualitatively.

Considering that post-hoc quantitative analyses fell more in line with qualitative responses, it may also be possible that confounding effects existed due to the heterogeneity of the presence of pets at home in the control group. While 73% of respondents reported having a dog as a pet, only 34% of respondents with dogs were registered to participate in the DAW program, meaning 64% of respondents with dogs did not bring their dog in but interacted with their dog outside of the workplace. Additionally, 12% of respondents had a pet that was not a dog and could not come to work at all. While statistically significant results were found in post-hoc analyses, these should be interpreted carefully to avoid Type I error. Rather than interpreting these as proof that dogs or other pets positively impact workplace wellbeing, they should be used as guidance in designing future studies of dogs in the workplace.

The fact that DAW participants choose when to bring their dogs to work may also be important to consider. Once registered, DAW participants are free to decide when and if they bring their dog to the workplace. Qualitative responses indicated not all DAW participants actually brought their dog to work or only occasionally brought their dog in, complicating understanding of the influence of dogs in the workplace. Barker et al. (2012) found, for employees that regularly brought their dog to work, they were significantly more stressed on days where they left their dogs at home.

Sample means were in line with population norms, indicating that neither DAW participants nor non-participants felt excessively unwell at work. In contrast, previous literature has demonstrated particularly low levels of workplace wellbeing in the veterinary field (Takefuji, 2025). It may be possible that the presence of dogs in the workplace in this study improves employee wellbeing generally, eliminating quantitatively measurable differences between groups while still allowing employee perceptions to manifest qualitatively (Hoffman et al., 2018; Gee et al., 2019; Clements et al., 2024). Additionally, Barker et al. (2012) found that employees of a company with a DAW program generally scored higher on perceived organizational support and job satisfaction compared to population norms, regardless of whether they brought a dog to work. It may be possible that the respondents’ general perception of their employer is similarly enhanced in our sample, across groups. This effect may be particularly salient in this study, due to the higher likelihood of employees in a veterinary setting caring strongly about dogs. This may lead to veterinary employees, regardless of their own pet status, caring strongly about their employer’s pet-friendly practices or lack thereof.

### Practical implications

A number of practical suggestions for improvement of DAW programs emerged from qualitative responses. These pertained to either the planning stage of implementing a DAW program, or to on-going maintenance of an established program. These practical suggestions can be implemented either by employers at the initial planning stages of implementing a DAW program, or when assessing pre-existing DAW programs. Developing comprehensive policies that anticipate and support employee needs is important when implementing pet-friendly practices, such as DAW programs (Wilkin et al., 2016). Additionally, workplace initiatives to address and prevent burnout were shown to have longer lasting effects when planning and implementation was shared between employees and employers (Wachter et al., 2020). Collaborative, thoughtful implementation of employee-support programs can serve as an environmental strength when the programs make employees feel safe, comfortable, and supported (Banyard et al., 2025; Whittenbury et al., 2025).

Employers can achieve this by incorporating employee feedback at all steps of the DAW program, from planning to on-going maintenance, and by allowing employees to be part of the planning and maintenance process (Foreman et al., 2017). Including employees as early as possible will also help employers include as many perspectives as possible and anticipate future roadblocks unique to their workplace, an aspect of implementing DAW programs where employers have previously struggled (Hall et al., 2017). Establishing a behavioral assessment, as seen in this DAW program, is strongly recommended to support safe interactions and minimize disruptions. Additionally, rules or guidelines of the program should be designed equitably for both those interested in participating and those who prefer not to interact with dogs (Wilkin et al., 2016; Hall et al., 2017; Pina e Cunha et al., 2019). Specifically, care should be taken to support access to participation for all interested employees. This can include considerations such as thoroughly announcing the program to all employees and addressing work factors outside of the employee’s control that may prevent participation. Employees that are uninterested in participation or unable to work near dogs should be accommodated through approaches such as establishing a “dog-free” area that employees can choose to work in. It can also be beneficial to consider alternatives for supporting wellbeing for those uninterested or unable to participate, such as flexible and remote working hours (Tables 3–5).

For currently running DAW programs, employers should actively monitor and improve the program (Wilkin et al., 2016; Foreman et al., 2017). To prevent workplace disruptions and unsafe human-dog or dog-dog interactions, periodic reassessments of behavior are recommended. The timing and rigor of these reassessments depends on each workplace’s unique context. Additionally, this study’s DAW program features a method to report dog-related concerns. When a concern is reported, a new behavioral assessment is conducted to determine if the dog is still appropriate for the program, if behavioral training is needed, or if the dog should be dismissed from the program. Utilizing the behavioral assessment in this manner allows for an objective on-the-spot assessment of dog behavior that supports safety and minimizes disruptions. To ensure that program guidelines are adhered to, an identifier should be utilized to indicate a dog has passed their behavioral assessment and is approved to participate. This can take the form of material attached to the dog’s leash, collar, or harness, a sign on office doors, or other signifiers as appropriate for a given workplace.

In addition to human concerns, dog welfare is a crucial consideration before and during implementation of a DAW program (Wells and Perrine, 2001; Foreman et al., 2017). The DAW program in this study operates in a workplace that is pet-friendly by nature, requiring less adjustment for the program to operate safely. Each workplace should thoroughly inspect both their physical environment and policies to ensure that dogs will be entering a safe setting. Specific considerations include choking hazards, machinery, dog fearfulness, zoonotic infections, and unsafe dog interactions (Foreman et al., 2017). Tools such as behavioral assessments and workspace audits can establish safe working environments for humans and dogs.

### Limitations

The results of this study should be considered within the context of the following limitations. This study is a cross-sectional exploratory study representing preliminary findings utilizing a small sample. As such, this study sets a foundation for future research on dogs in the workplace, particularly structured long-standing DAW programs, but cannot determine causality, and outcomes cannot specifically be attributed to bringing dogs into the workplace.

Individual workplace factors, such as the specific work role, number of clinical hours, or type of office may have served as a moderator in this sample. While these factors were measured in this study, the cross-sectional design prevents conclusions on the nature of their effects. Additionally, the presence of dogs and other pets at home for respondents in the control group may have acted as a confounding variable. Other unmeasured variables, such as the DAW dog’s behavior and personality traits, or level of attachment between the dog and human, could also have affected findings. A longitudinal study of a DAW program before, during, and after implementation, with a large sample size, would serve to address these limitations and support a more thorough understanding of the effects of dogs in the workplace.

The sample in this study was drawn from a small group of employees at one veterinary college. The small size of the college consequently limited the sample size. It is possible that this contributed to the mismatch between quantitative and qualitative findings. The sample size was sufficient for qualitative analysis, but may have led the quantitative analysis to be underpowered. Additionally, this program is being utilized by employees in a specialized field, specifically staff and faculty within one veterinary college. As workplaces settings vary widely, generalizability is limited. Future studies should evaluate DAW programs within a variety of workplaces, especially in settings that are not already animal-oriented.

### Future directions

This study explored perceptions of a well-established bring-your-dog-to-work program that has run for multiple decades. The respondents demonstrated a higher-than-expected level of perceived workplace wellbeing regardless of their participation in the DAW program. To better understand the impacts resulting specifically from a bring-your-dog-to-work program, research should be conducted in partnership with an employer looking to implement this type of program for the first time. Future studies should also consider incorporating physiological measures of stress and wellbeing alongside measures of perceived wellbeing for both the humans and animals involved. Additionally, in this study, meaning-making strengths were present in quantitative findings but did not emerge in correlation to the DAW program qualitatively, despite previous research linking pet dogs to identity and purpose. Further investigation is warranted to understand how meaning-making occurs in the veterinary workplace, and how dogs in the workplace may or may not influence work-related meaning-making.

## Conclusion

Resilience can help employees mitigate the challenges of high-stress work environments and enhance employee wellbeing. This study found that employees at a midwestern veterinary school possessed multiple strengths within the RPM framework, representing all four strength categories. By employing the RPM framework to examine employee wellbeing in a veterinary setting, this study contributed to the understanding of resilience in high-stress workplaces.

This study was the first to apply the RPM framework to human-animal interaction, specifically a long-standing DAW program at a veterinary college. Qualitatively, respondents indicated that bringing their dogs to work supported their physical, mental, and social wellbeing. Quantitative findings did not show meaningful differences between those that participated in the program and those that did not. However, it is notable that respondents demonstrated higher resilience and wellbeing than expected for the veterinary field, regardless of their participation in the DAW program. This may indicate that employees at this veterinary college are generally highly resilient. Further investigation is warranted to understand this population’s resilience, and to understand the relationship between resilience and bringing your dog to work.

Additionally, perceived benefits of the DAW program appeared to outweigh potential challenges. Respondents highlighted important recommendations for employers looking to implement their own bring-your-dog-to-work programs, including employee buy-in and periodic behavioral reassessments. Developing effective and well-designed bring-your-dog-to-work programs should continue to be investigated as a potential method of supporting employee wellbeing and resilience.

## Data Availability

The datasets presented in this article are not readily available because the data may be identifying to the participants. Requests to access the datasets should be directed to Leanne Nieforth, lniefort@purdue.edu.
