# Supplementary material for: A dogs-at-work program in a veterinary college: promoting workplace wellbeing and resilience
Source: Front Psychol. 2026 Mar 26;17:1768459. doi: 10.3389/fpsyg.2026.1768459 (PMC13062298; doi:10.3389/fpsyg.2026.1768459)
Supplement: Supplementary file 1 [file Data_Sheet_1.PDF]

Dogs-At-Work Survey Questions

Questions have been edited for confidentiality.

1. Are you staff, faculty, graduate student staff, postdoc staff, other?
    a. You selected “other” for your position. Please list your position.
2. What is your role in the college?
3. Do you have clinical duties in the hospital?
    a. On average, how many days a week do you work in the hospital?
4. How many years have you been in your role?
5. Do you have a shared or a private office?
6. How many days of leave (personal, family or pet-related) have you taken in the past year?
7. Do you have pets?
    a. How many days have you taken off work due to your duties as a pet owner in the past year?
    b. Do you have a dog?
        i. The [Dogs-At-Work (DAW)] Program is a bring your dog to work program available at [Veterinary College]. To be registered as a [DAW] Dog, pet dogs must be evaluated by a member of the behavior team. If the dog passes the evaluation, they can be registered as a [DAW] Dog. Are you familiar with this program?
        ii. Do you bring your dog to work?
            1. On average, how many days per week do you take your dog to work?
                a. What determines this number?
            2. How does your dog interact with others (human or animal) in the College?
            3. What is your motivation (or reasons) for bringing your dog to work?
            4. Do you have a [DAW] Dog?
                a. Describe your overall experience with the [DAW] program.
                b. How satisfied are you with the [DAW] Program on a scale of 1-10?
                c. What is the reason that you have not registered your dog as a [DAW] Dog?
                d. What determines when you bring your dog versus when you do not bring your dog?
8. Have you interacted with a registered [DAW] Dog?
    a. Describe your overall experience with the [DAW] Dog program.
9. Is there anything you would change about the [DAW] Program?
    a. What would you change about the [DAW] Program?
10. What your thoughts on the [DAW] program?
11. Is there anything else that you would like to share with the research team?
